# Cold Disinfestation of *Zeugodacus tau* (Diptera: Tephritidae) on Oranges Using Artificial Infestation Method

**DOI:** 10.3390/insects17030335

**Published:** 2026-03-19

**Authors:** Jiajiao Wu, Mutao Wu, Lixia Feng, Weisong Li, Zhihong Li, Qiang Xu, Haijun Liu, Tao Liu, Sihua Yang

**Affiliations:** 1 Guangzhou Customs District Technology Center, No. 66 Huacheng Avenue, Tianhe District, Guangzhou 510623, China; wujj@iqtcnet.cn (J.W.); wumt@iqtcnet.cn (M.W.); fenglx@iqtcnet.cn (L.F.); xuqiangbj@foxmail.com (Q.X.); yangsh@iqtcnet.cn (S.Y.); 2 Department of Plant Biosecurity, College of Plant Protection, China Agricultural University, Beijing 100193, China; keertraplll_ws@163.com (W.L.); lizh@cau.edu.cn (Z.L.); 3 Chinese Academy of Quality and Inspection & Testing, Beijing 100123, China

**Keywords:** cold tolerance, phytosanitary treatment, *Zeugodacus tau* Walker, fruit fly, orange

## Abstract

*Zeugodacus tau*, an economically important fruit fly species, exhibits a preference for infesting the fruits of cucurbitaceae, but it has also been reared from the fruits of several other plant families. Phytosanitary treatments are needed to ship the fruit from some of these host plants out of areas where populations of the fruit fly exist. Based on the guidelines for the development of cold disinfestation treatments for fruit fly host commodities, proposed by the International Plant Protection Convention (IPPC), the cold disinfestation trials were carried out with *Z. tau* infesting oranges through artificial infestation. The results showed that the third instar of *Z. tau* was the most tolerant stage among all developmental stages. No survivors were found among 106,204, 96,168, and 9180 individuals of *Z. tau* in oranges treated at 1.75 °C for 23 d, 2.34 °C for 25 d, and 1.8 °C for 22 d, respectively. These results support for the application of the 22-day treatment at ≤1.67 °C as an additional safety measure. Such a measure mitigates the risk of introduction and establishment of *Z. tau* through imported citrus. However, for other susceptible hosts of *Z. tau*, the cold treatment schedules against *Z. tau* required to achieve quarantine security with larval endpoint would need to be 23 d at 1.75 °C or 25 d at 2.34 °C. These results also indicated that *Z. tau* exhibits the highest cold tolerance among the other tephritid species for which cold treatment have been reported.

## Simple Summary

Cold treatment has been employed since the early 20th century to ensure phytosanitary control of fruit flies in various fruits. A cold treatment protocol for *Zeugodacus tau* must be developed to improve global fruit trade. Therefore, cold disinfestation trials were carried out with *Z. tau* infesting oranges through artificial infestation. There were no survivors among 9180 individuals of *Z. tau* in oranges treated at 1.8 °C for 22 days. This data provided support for the application of the 22-day treatment at ≤1.67 °C as an additional safety measure. Such a measure mitigates the risk of introduction and establishment of *Z. tau* through imported citrus. However, for other susceptible hosts of *Z. tau*, the cold treatment schedules need 23 d at 1.75 °C or 25 d at 2.34 °C with larval endpoint to achieve quarantine security. These results will provide sufficient evidence for the development of phytosanitary treatments against *Z. tau* under ISPM 28, thereby facilitating trade in its host fruits.

## 1. Introduction

*Zeugodacus tau* Walker (pumpkin fruit fly) was first reported by Walker (1849) from China and is now found in the Oriental region [1]. The fruit fly exhibits preference for infesting the fruits of cucurbitaceae, but it has also been reared from the fruits of several other plant families, including Anacardiaceae, Fabaceae, Lithomyrtus, Moraceae, Passifloraceae, Sapotaceae, Solanaceae and Rutaceae [2,3,4]. The invasion of fruit flies is influenced by various factors, such as phenotypic plasticity of thermal tolerance [5]. Phytosanitary treatments are needed to ship the fruit from some of these host plants out of areas where populations of the fruit fly exist. For example, citrus exported from China to the United States is required to undergo cold treatment against fruit flies including *Z. tau* [6]. Cold treatment, a non-chemical, safe, and effective method, has been employed since the early 20th century to ensure phytosanitary control of fruit flies in various fruit cultivars cultivated worldwide [7]. Research data have demonstrated that cold treatment on citrus or other fruits such as kiwifruit at the temperature of ≤3 °C is an effective approach to managing fruit species including *Ceratitis capitata*, *Bactrocera zonata* and *B. dorsalis* [8,9,10,11,12]. An increasing number of cold treatment schedules on fruit flies have been accepted as international standards, i.e., phytosanitary treatment No. 16 (PT 16) for *B. tryoni*, PT 24 for *C. capitata* and PT 41 for *B. zonata*, all listed in International Standards for Phytosanitary Measure No. 28 (ISPM 28) [13,14,15]. At the time of writing, the only available PT for *Z. tau* is irradiation at doses of 72 Gray and 85 Gray accepted as PT 42 of ISPM 28 [16]. Published studies on cold tolerance of *Z. tau* are limited, and the existing results indicate a few days’ differences on exposure time under similar cold treatment temperature across different fruits. For example, Dia et al. reported that a 22 d phytosanitary cold treatment at ≤ 1.70 ℃ did not prevent four larvae (out of over 40,000 estimated individuals) of *Z. tau* from developing to the puparial stage [17]. In contrast, Prakriti et al. reported that a 15 d phytosanitary cold treatment at 1–1.5 °C on cherry tomatoes, designed to eliminate the most cold-tolerant stages of *Zeugodacus cucurbitae*, can effectively eliminate all development stages of *B. dorsalis* and *Z. tau*, which exhibited lower cold tolerance levels [18]. Two cold treatment schedules, T107-n and T107-o, are included in the United States Department of Agriculture (USDA) Treatment Manual. The schedule T107-n (≤1 °C for 17 d) is the only stand-alone cold treatment schedule currently available for *Z. tau* and applies only for guavas. The schedule T107-o (18, 20, and 22 d at ≤0.56, 1.11, and 1.67 °C, respectively) can only be used as part of a systems approach, which includes registered places of production, trapping requirements, and grove sanitation, to treat Chinese citrus (not including bagged pomelo) for export to the USA [17]. The scarce number of approved phytosanitary treatments for *Z. tau* and published studies, combined with its high dispersal capacity and invasive potential [19,20,21], pose a serious threat to agricultural production systems [17]. Further research for the cold tolerance of this economically important fruit fly, used as phytosanitary treatments, is essential.

Host preference and suitability tests have shown that citrus is less susceptible to *Z. tau* than cucurbit fruit under laboratory and field conditions [3,4]. However, *Z. tau* was listed as a quarantine concerned species on some international citrus trades, such as the exportation of Chinese citrus for the USA [6]. Also, the schedule T107-o can only be used as part of a systems approach to treat citrus (not including bagged pomelo) from China to the USA. The application of the treatment schedule of the 22 d at ≤1.70 ℃ (one of schedules of T 107-o) for mitigating the risk of *Z. tau* introduction and establishment through the imported citrus pathway was supported by Dia et al. [17]. However, any live larva may pose a challenge for quarantine inspection, as cold treatments are typically proposed based on efficacy data targeting acute larval mortality as the treatment endpoint [22]. Thus, any live life stages of regulated fruit fly species found post-treatment would be considered a treatment failure [13,14,15].

Owing to the insufficient research on cold treatment for *Z. tau*, studies targeting larval endpoint are required to meet the requirements of quarantine treatment standards. In this study, cold treatment trials were conducted in accordance with the guidelines developed by the International Plant Protection Convention (IPPC) for establishing cold disinfestation treatments for fruit fly host commodities [22]. These results will provide sufficient evidence for the development of phytosanitary treatments against *Z. tau* under ISPM 28, thereby helping regulatory authorities in importing countries establish appropriate cold treatment schedules for *Z. tau* and facilitating trade in its host fruits.

## 2. Materials and Methods

### 2.1. Test Insects

The *Z. tau* populations were obtained from pumpkin (*Cucurbita moschata* Duch) planted for non-commercial production in Huadu district of Guangzhou city (latitude 24.1956° N, longitude 120.7105° E), Guangdong, China, during the years 2022–2024. These insects were maintained at the Plant Quarantine Institute of Guangzhou Customs District Technology Center in Guangzhou, Guangdong, China, and were reared and reproduced for this species during different trial seasons. The methods of rearing and reproduction are as follows: The baby pumpkin fruits infested by fruit fly (2–4 kg in each trial season) were collected and kept in the plastic tray lined with paper towel at the base, covered with insect-proof mesh for the larvae development in laboratory. Late third instar larvae that jumped out of pumpkin were transferred to a moist sand-filled jar for pupariation. Before adult eclosion (about 5–6 d age of pupae), the pupae (200–300 individuals) were put in an aluminum cage (58 × 40 × 40 cm) with three sides covered by mesh-type organza. Once the emergence took place, the adults were identified as *Z. tau* (no other species of fruit fly was found in pumpkin fruits during our collections from the field) and fed with a diet of one part solid hydrolyzed yeast and three parts white sugar. Water for the adult fruit flies was provided through a water-soaked sponge. Some slices of fresh orange were supplied occasionally. Eggs were collected by placing a pumpkin slice (c. 0.2 kg) in cage housing gravid adults (c. 3 weeks-old) of fruit fly for 4–6 h to allow egg oviposition. The pumpkin slice with eggs was transferred to a plastic tray filled with paper, covered with insect proof mesh, and then maintained at 26 ± 2 °C and 70 ± 5% relative humidity (RH) for egg hatching and larvae development to late-aged third instar (5–7 d). Fresh pumpkin slices were supplied in time for feeding larvae. Once the growing larvae reached the mature third instar stage, the plastic tray was moved to a moist sand-filled container (40 × 30 × 18 cm) for the larvae to naturally jump out and then pupariate. Pupae were collected from the container, and 4000 to 5000 pupae were placed inside another rearing cage until adult emergence. The same methods were replicated until the number of adults was enough for testing. The colony consisted of 8000–20,000 adults (2–4 cages each containing 4000–5000 flies, with sex ratio of 1:1), according to the testing required, and was maintained at 26 ± 2 °C and 70 ± 5% RH in a dark–light cycle of 14:10 h. The fruit fly colonies had been maintained for nearly 6 months (August to January of following year) of each trial season. Trials were conducted using colonies of about 3–5 generations.

### 2.2. Test Fruits

Oranges (*Citrus sinensis* (L.) Osbeck) were chosen for the trials due to their long period availability and their lower susceptibility to cold treatment compared to other citrus varieties [12]. The oranges with a consistent maturation grade and free of defects were sourced from Jiangxi Hongyuan Fruit Co. Ltd., Fuzhou, China, and were kept in a cooling incubator set at temperatures ranging from 4 to 5 °C and 70 ± 5% RH until use. Before the cold assays (c. 20 h), the fruits (mean weight = 209.50 ± 13.01 g for Navel and 185.50 ± 12.79 g for Valencia) were moved to a storage room and covered with insect-proof mesh for the fruit temperature to recover to ambient temperature. Both Navel and Valencia cultivars of orange were used in large-scale trials, while only Navel orange was used in other trials.

### 2.3. Test Facilities

Cold treatment of infested oranges was conducted in a cold treatment chamber (HLT103PB) supplied by Chongqing Well Zhenchang Technology Co., Ltd., Chongqing, China, which has a capacity of 3.15 m^3^ (inside dimensions: 1.4 × 1.5 × 1.5 m). The facility installed a dry air system to ensure no frost forms inside the chamber during the whole period of cold treatment trials [12]. The temperature recording system is implemented with midi Logger GL840 (GRAPHTEC Corporation, Yokohama, Japan, ver. 1.31), which has 10 temperature sensors (Pt 100, A grade) for chamber environment and fruit and a sensor for chamber humidity. Two cooling incubators, Climacell Bzv, CLC 222 and CLC 707 from MMM Groups, München, German, which boast a stated accuracy of ±0.5 °C, were used. The internal dimensions are 0.52 × 0.54 × 0.76 m for the CLC 222 and 0.52 × 0.94 × 1.45 m for the CLC 707, both featuring temperature and humidity controls.

### 2.4. Fruit Infestation

**Figure 1:**
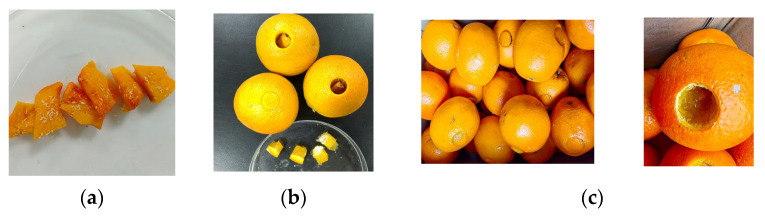
Main steps of the artificial infestation method with the hollowed-out fruit pulp: (**a**) pumpkin pieces with eggs; (**b**) pulp hole with eggs/pumpkin pieces and its fruit plugs; (**c**) infested oranges ready for cold treatment (**left**) and the third instar larvae in the infested orange (**right**).

Test fruits were removed from the cooling incubator and equilibrated at 26 ± 2 °C and 70 ± 5% RH for 24–28 h to ensure suitable conditions for larval development. The method of artificial infestation of *Z. tau* on orange was carried out with the hollowed-out fruit pulp method described by Wu et al. [12]. The method was modified as follows (see Figure 1): Eggs of the fruit fly were collected with pumpkin slices for 2–3 h, which were then placed in the incubator (CLC 222) at 22 °C or 25 °C and 65 ± 5% RH. Prior to egg hatching (24–28 h for 22 °Cor 16–18 h for 25 °C), the pumpkin slices were cut into small pieces (c. 10 × 20 mm). For each test orange, a hole was made through the rind and pulp to the fruit center (c. 30 mm) using a cork borer (19 mm diameter, size 11), and the fruit plug with orange rind and its pulp were removed. Then, one or two pumpkin pieces containing eggs were put inside the hole, and the hole was sealed with the fruit plug (a portion of the pulp was removed from the plug with sterile scissors to accommodate the egg-pumpkin pieces)**.** Infested oranges were arranged in a labeled plastic basket with dimensions of 40 × 30 × 12 cm, then replaced back in the incubator at 22 °C or 25 °C and 65 ± 5% RH for larvae development. The oranges were not treated with any chemical and disinfectant because very few oranges after artificial infestation of *Z. tau* using the hollowed-out fruit pulp method were contaminated by microorganisms during our trials.

### 2.5. Evaluation of Infested Fruits

The evaluation of infested orange was carried out by the method of washing fruit pulp. The method was described by Wu et al. [12], and modified procedures were listed as follows: The treated and control fruits were put in a plastic basket (35 × 27 × 10 cm) with hole sizes of about 3 mm × 5–10 mm to facilitate larvae moving or sinking down easily and keep the large tissues of orange in the basket. No more than ten oranges of each basket were evaluated at the same time. Subsequently, the basket with fruits was dipped into a plastic container (38 × 28 × 12 cm) filled with clean water containing approximately 70% of the container’s volume. Then, the fruit pulp tissues were manually dissected and washed. After a few minutes, the basket with tissues was removed and placed in another container containing the same volume of water. The pupae and live larvae in the first container were collected and recorded. The processes of dissecting, washing, collecting and recording any pupae and live larvae were repeated. This procedure was continued until two consecutive repeated processes yielded no survivor (repeated 4–5 times). Larvae that pupated were considered survivors, and any larvae showing movement were classified as alive [10]. Larvae that remained motionless were considered dead. The status of any unresponsive larvae was assessed by placing them on a napkin to observe if they would exhibit any signs of movement.

### 2.6. Development of Immature Stages of Fruit Flies in Orange

**Figure 2:**
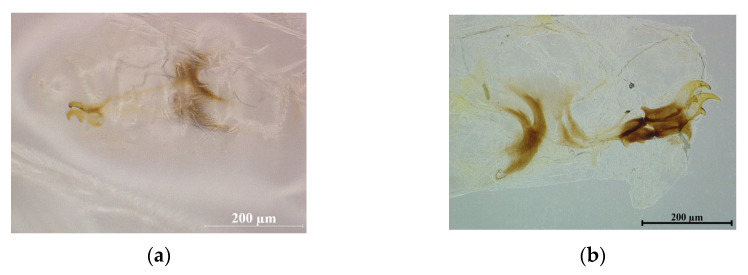

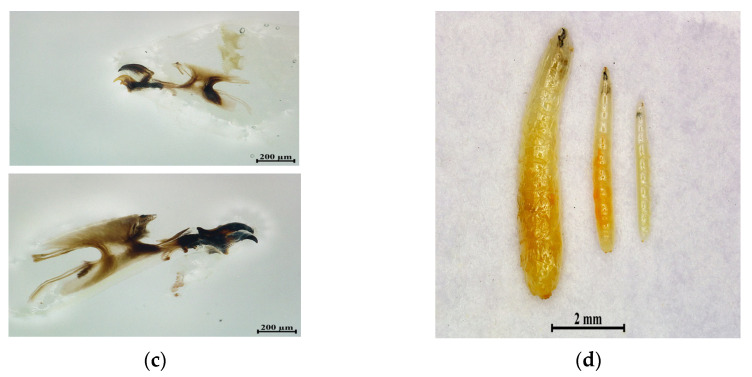
Cephalopharyngeal skeleton characteristics and size of different instar larvae of *Zeugodacus tau*: (**a**) first instar: slender oral hooks and undeveloped pypopharyngeal sclerites; (**b**) second instar: more developed pypopharyngeal sclerite and stronger oral hooks with preapical teeth; (**c**) third instar: new cephalopharyngeal skeleton and its old one together ((**up**): early third instar) and deeper color pypopharyngeal sclerites with darker color oral hooks without preapical teeth (**down**); (**d**) size of larvae (left: third instar, middle: second instar, right: first instar).

The oranges after artificial infestation were placed in a cooling chamber (CLC 222) at 22 °C or 25 °C and 65 ± 5% RH for various periods to evaluate the impact on their development into various larval stages. At least three oranges were randomly selected and dissected every day for 8 d (22 °C) or 6 d (25 °C) to check the life stage present and the number of insects of each developmental stage. Over 100 larvae were examined during each period using a stereoscope to assess the development of the larvae. The experiment was repeated three times. Since the larval characteristics of *Z. tau* had been described by Singh et al. and Prakriti et al. [1,18], we showed the larval cephalopharyngeal skeleton with its oral hock of each instar of *Z. tau* and its size of different instar (see Figure 2). The highest significant difference among the instar stages is that the first instar has slender oral hooks and undeveloped pypopharyngeal sclerites, which are located at the base of the oral hooks and connected to each other. In contrast, the second instar and third instar have stronger oral hooks and more developed pypopharyngeal sclerites (darker color). Specifically, the second instar has oral hooks with preapical teeth, whereas the third instar has darker color oral hooks that exhibit vestigial preapical teeth.

### 2.7. Most Cold-Tolerant Life Stage Trial

Different developmental stages of *Z. tau* in Navel oranges were exposed to the cold treatment chamber for various periods to examine the effect of cold disinfestation on pest mortality. The eggs/pumpkin slices and, following that, the oranges after artificial infestation were placed in the incubator (CLC 707) and incubated at 22 °C and 65 ± 5% RH for 1, 2, 4, and 6 d (including 1 d of eggs in pumpkin slices incubated before being inoculated into orange) to allow the development of eggs or majority of larvae to the first, second, and third instar, respectively, based on the results from development rate trials (see Table 1). Three replications were conducted separately.

Each control and treatment group contained 60 infested oranges (10 serving as controls) for each life stage, including eggs (1 day-age) and first, second, and third instar. Before being loaded into the cold treatment chamber, ten infested fruits from the same developmental stage were selected randomly and placed in one labeled insect-proof mesh bag. Subsequently, the 4 bags which contained fruits infested with different life stages were grouped together and placed in one carton (40 × 36 × 22 cm) for convenient removal from the treatment chamber based on the exposure time (0, 3, 6, 9, 12 and 15 d). The oranges of the control group were kept at 25 °C for 6, 5, 3, and 1 d in accordance with their development stage (eggs and first, second, and third instar, respectively) for developing to large third instar and then evaluated to determine the average number of survivors of each fruit according to the methods of washing fruit pulp described previously.

One of the cartons with 4 bags of infested oranges was then removed from the cold chamber at intervals of 3, 6, 9, 12 and 15 d and kept in an incubator (CLC 707) with the temperature of 25 °C for 6, 5, 3, and 1 d in accordance with their development stage (eggs and first, second, and third instar, respectively). Then, the oranges with survivors developed into third instar were dissected and evaluated to observe the mortality rate of each trial. The number of effective larvae used in the treatment group was calculated based on the number of survivors in the control group [20]. During the experiment, temperatures were recorded every 60 min with midi Logger GL840. Three pulp temperature sensors were inserted in the core of the orange fruit (the larger one in each trial) and placed in a diagonal pattern. The average time (mean ± SD) for the oranges to cool down to the target temperature of 1.8 °C or 2.5 °C was 20.67 ± 2.08 h or 17.0 ± 2.65 h, respectively.

### 2.8. Small-Scale Trial

The previous most cold-tolerant life stage trials showed that the third instar larvae of *Z. tau* exhibited the highest cold tolerance both at 1.8 °C and 2.5 °C (see Table 2); therefore, further experiments were conducted on this species at third instar larva. Artificial infestation was carried out with the hollowed-out fruit pulp method as previously described. Eggs/pumpkin pieces of *Z. tau* were inoculated into 180 Navel oranges, of which 30 were control oranges and 150 were test oranges. The oranges were placed in a CLC 707 incubator with a temperature of 22 °C and RH of 65 ± 5% for 6 d, at which time the eggs had grown into third instar. Before treatment, the 150 test oranges were packed in five cartons (36 × 28 × 22 cm), placed inside the cold chamber, and chilled until their core temperatures were 1.8 °C or 2.5 °C. They were kept in the cold chamber for 17, 19, 21, 22 and 23 d at 1.8 °C (for 19, 21, 23, 24 and 25 d at 2.5 °C). The control infested oranges were packed in one carton, enclosed in an insect-proof mesh bag, and kept at 25 °C for 24 h before evaluation. After each cold treatment duration, one carton of the infested oranges was removed and kept in an incubator with a temperature of 25 °C and RH of 65 ± 5% for 24 h to allow the fruit flies to regain mobility. Then, the oranges were dissected and evaluated. The mortality rate of the fruit flies was determined based on the number of larvae in the control group [22]. During the experiment, temperatures were recorded every 60 min with midi Logger GL840. Three pulp temperature sensors were inserted in the core of the orange fruit (the larger one in each trial) and placed in a diagonal pattern. Three replications were performed separately for Navel orange both at 1.8 °C and 2.5 °C. The average time (mean ± SD) for the oranges to cool down to the target temperature of 1.8 °C or 2.5 °C was 20.33 ± 1.53 h or 17.0 ± 1.73 h, respectively.

### 2.9. Large-Scale Trial

Based on the result of small-scale trials, the optimal treatment duration, which reached two consecutive 100% mortality, was adopted as criteria to determine the treatment duration of the large-scale trial [22]. Three replications were performed separately for Navel and Valencia cultivars of oranges at the temperature of 1.8 °C and 2.5 °C. Artificial infestation was carried out with the hollowed-out fruit pulp method as previously described. Egg/pumpkin pieces of *Z. tau* were inoculated into 250 oranges of each variety, of which 50 were control oranges and 200 were test oranges. The oranges were kept in an incubator (CLC 707) with a temperature of 22 °C and RH of 65 ± 5% for 6 d, at which time the inoculated eggs had grown into third instar. The test oranges (80%) were packed in 12 cartons (36 × 25 × 22 cm) before treatment. Each carton stored 15 or 17 infested oranges (10 cartons for 17 oranges and the other 2 cartons for 15 oranges) and was supplemented with non-infested oranges of the same cultivar to fill the boxes. Additionally, 24 boxes of non-infested oranges of the same cultivar were also placed inside the cold treatment chamber. A total of 36 fruit boxes were randomly grouped into 12 columns and 3 rows. The other 20% of infested fruits were packed in 2 cartons as the control group [22], enclosed in an insect-proof mesh bag, and kept at 25 °C for 24 h before evaluation. Six pulp sensors were inserted in the core of the orange fruit (the larger one in each trial) without infestation and were strategically positioned across different rows and sites in a diagonal pattern for each replication. According to the guidelines for the development of cold disinfestation treatments for fruit fly host commodities proposed by the IPPC, the treatment was deemed to have started when half the fruit pulp sensors reached the treatment temperature [22]. Temperature recordings were automatically logged at 60 min intervals throughout the trial. All temperature sensors were calibrated in melting ice using a certified mercury glass thermometer before each trial to verify their accuracy. The average time (mean ± SD) for the oranges to cool down to the target temperature of 1.8 °C or 2.5 °C was 22.33 ± 2.52 h or 28.67 ± 1.53 h, respectively.

At the end of the treatment, after 23 d at 1.8 °C or 25 d at 2.5 °C, the cartons containing infested fruits were removed from the cold treatment chamber and fully enclosed with insect-proof mesh bags. They were stored in an incubator at 25 °C for 24 h before evaluation. The effectiveness of the treatment was gauged by calculating the number of larvae in the treatment group relative to the number of surviving larvae in the control group [22].

### 2.10. Statistical Analysis

In all trials, the number of effective individuals used in the treatment group was calculated based on the number of survivors in the control group (20%). To evaluate the cold tolerance of various life stages, time-response mortality data for *Z. tau* were subjected to probit and logit analysis using the PoloPlus (Version 2.0), LeOra software, Berkeley, CA, USA. This statistical analysis was applied to the data collected throughout this study to determine the predicted exposure periods needed to reach 99% (LT_99_) and 99.9968% (LT_99.9968_) mortality. The lethal time (LT) data according to the results of probit and logit analysis also was analyzed with one-way analysis of variance (ANOVA) followed by Tukey’s multiple range test (*p* < 0.05) using IBM SPSS Statistics 19.0.

## 3. Results

### 3.1. Larval Development Rate of Z. tau in Orange

The percentages of *Z. tau* at different growth stages dissected from infested orange after incubation at 25 °C or 22 °C are shown in Table 1. During the incubation at 25 °C, the eggs did not hatch within 0.7 d and began hatching at approximately 0.9 d; the majority of eggs (93.22%) hatched for 1 d incubation, and more than half the larvae (59.91%) developed into the second instar by day 2. The highest number of the second instar (83.97%) was observed on day 3, and by days 4 and 5, the percentages of third instar were 83.04% and 96.81%, respectively. According to this result, the developmental periods required for *Z. tau* eggs in oranges at 25 °C to develop into the first, second, and third instar with over 83% of individuals reaching the target stage were 1 d, 3 d, and 4 d, respectively. At the temperature of 22 °C incubation trial, the eggs did not hatch after 1 d incubation and began hatching at approximately 1.5 d; the majority of eggs (95.9%) hatched into the first instar by day 2. The proportion of second instar increased on days 3 and 4, reaching 95.44% by day 4. About 30% of the insects remained at the second instar on day 5, while 94.43% developed into the third instar by day 6. The results showed that the developmental periods required for *Z. tau* eggs in oranges at 22 °C to develop into the first, second, and third instar with over 94% of individuals reaching the target stage were 2 d, 4 d, and 6 d, respectively. In summary, both intervals of 0.7, 1, 3, and 4 d for the eggs developed to relevant stages at 25 °C incubation and that of 1, 2, 4 and 6 d at 22 °C incubation can meet the requirements of majority numbers (more than 83% at 25 °C or 94% at 22 °C, respectively) in the relevant developmental stage. Regarding the convenience of trial arrangement, 22 °C was selected as the incubation temperature for subsequent trials.

### 3.2. Effect of Most Tolerant Stage Trials

The results showed that the mortality of the eggs and larvae in infested oranges increased rapidly with the increase in cold exposure time both at 2.5 °C and 1.8 °C (see Table 2). The average temperatures (mean ± SD) of three replications were 2.54 ± 0.36 °C and 1.81 ± 0.23 °C. At both temperatures, 100% mortality of the eggs in Navel orange was observed by day 12, with the first instar larvae by day 15. However, by day 15, the mortality of second instar and third instar larvae were 99.36% and 98.35%, respectively, at 1.8 °C, with similar results (99.81% and 96.93%) for the 2.5 °C trials. The data demonstrated that the third instar larva exhibited the highest cold tolerance among all developmental stages at both cold treatments of 1.8 °C and 2.5 °C. The differences of mortality were significant at the 95% confidence level (CL) between the third instar and second instar for all exposure times at 2.5 °C. However, no significant differences (*p* > 0.05) in mortality were observed at 1.8 °C until the exposure time reached 15 d.

The parameters from the results of the analysis with probit and logit models, including slope and estimated time of cold treatment required to produce 99% lethal time (LT_99_) and 99.9968% (LT_99.9968_) mortality at 95% CL, are presented in Table 3. As shown, the amount of exposure time necessary for the third instar larvae under LT_99_ or LT_99.9968_ was longer than that for the other stage at both cold treatments of 1.8 °C and 2.5 °C, indicating that the third instar larvae have the highest cold tolerance. For the 1.8 °C trials, the differences of LT_99_ or LT_99.9968_ were not significant (*p* > 0.05) between the third instar larvae and second instar larvae, but both stages of larvae showed significant differences (*p* < 0.05) compared with the egg or the first instar stage. For the 2.5 °C trials, the differences of LT_99_ or LT_99.9968_ were significant (*p* < 0.05) in third instar larvae compared with the other life stages. Thus, the third instar larvae were determined to be the most tolerant stage. Hence, the following small-scale and large-scale confirmatory trials were performed only with third instar larvae. Furthermore, based on the probit analysis, the maximum estimated cold treatment lethal times of the most tolerant stage (third instar larvae) under LT_99.9968_ at 95% CL were 23.1 d and 24.8 d at 1.8 °C and 2.5 °C, respectively. This result was used for selecting the treatment periods for small-scale trials.

### 3.3. Small Scale Trials

In the small-scale trials, the infested oranges with third instar larvae of *Z. tau* were exposed to the cold chamber for 17, 19, 21, 22 and 23 d at 1.8 °C (the average temperature (mean ± SD) of three replications was 1.83 ± 0.34 °C) and for 19, 21, 23, 24 and 25 d at 2.5 °C (the average temperature (mean ± SD) of three replications was 2.47 ± 0.21 °C), respectively, to achieve 100% mortality. For the trials conducted at 1.8 °C (see Table 4), the third instar larvae exhibited mortality rates exceeding 99.50% by days 17 and 19. Three live larvae among 9108 individuals were found in one of three replications by day 21, and the mortality rate reached 99.96%. For all 9108 individuals, 100% mortality was observed both by days 22 and 23. Subsequently, a 23 d cold treatment duration was selected for the confirmatory trial of 1.8 °C. For the trials conducted at 2.5 °C (see Table 5), the third instar larvae exhibited mortality rates exceeding 99.09% by days 19 and 21. Two live larvae among 6399 individuals were found in one of three replications by day 23, and the mortality rate reached 99.96%. For all 6399 individuals, 100% mortality rate was observed both by days 24 and 25. Subsequently, a 25 d cold treatment duration was selected for the confirmatory trial of 2.5 °C.

### 3.4. Large-Scale Trials

After obtaining two consecutive instances of 100% mortality in the small-scale trials (see Table 4 and Table 5), the treatment periods in large-scale trials were selected for 23 d at 1.8 °C and for 25 d at 2.5 °C, respectively. The average number (mean ± SD) of third instar larvae per orange from the control groups was 95.0 ± 36.86 for Navel and 90.7 ± 23.57 for Valencia as well as 78.0 ± 17.78 for Navel and 80.0 ± 18.36 for Valencia at 1.8 °C and at 2.5 °C, respectively. This result showed that the artificial infestation of *Z. tau* on oranges with the hollowed-out fruit pulp method was confirmed as practical. This method could obtain a sufficient number of individuals during cold treatment trials even though oranges are considered less susceptible to *Z. tau*.

The results found in the large-scale disinfestation trials are presented in Table 6 and Table 7. In the trials conducted at 1.75 °C (the minimum average temperature of six replications of two cultivars), the temperatures of three replications were 1.88 ± 0.10 °C, 1.82 ± 0.12 °C and 1.75 ± 0.11 °C for Navel orange, respectively, and 1.80 ± 0.09 °C, 1.76 ± 0.11 °C and 1.79 ± 0.08 °C, for Valencia orange, respectively. The exposure times exactly of three replications were 22.75, 22.88 and 22.91 d for Navel orange, respectively, and 22.79, 22.83 and 22.91 d for Valencia orange, respectively. The estimated total number of larvae in the treated oranges during the large-scale trials was 106,204 (57,296 from Navel orange and 48,908 from Valencia orange). After a 23 d treatment at 1.75 °C, no survivors were found among the treated oranges, indicating a 100% mortality for over 100,000 individuals of the most cold-tolerant stage of the *Z. tau* in oranges (see Table 6). Both Navel orange and Valencia orange treated at 1.75 °C for 23 d against *Z. tau* exceeded the required minimum assurance of 99% mortality at the 95% CL. Cold treatment of *Z. tau* on oranges for combined Navel and Valencia cultivars at 1.75 °C for 23 d exceeded the required minimum assurance of 99.9968% mortality at the 95% CL and also passed the probit-9 level.

In the trials conducted at 2.34 °C (the minimum average temperature of six replications of two cultivars), the temperatures (mean ± SD) of three replications were 2.61 ± 0.15 °C, 2.43 ± 0.13 °C and 2.56 ± 0.13 °C for Navel orange, respectively, and 2.54 ± 0.18 °C, 2.34 ± 0.23 °C and 2.53 ± 0.17 °C for Valencia orange, respectively. The exposure times exactly of three replications for Navel orange were 24.88, 24.79 and 24.75 d, respectively, and 24.79, 24.67 and 24.83 d for Valencia orange, respectively. The number of estimated larvae in the treated oranges during the large-scale trials was 96,168 (50,768 from Navel orange and 45,400 from Valencia orange). No survivors were detected in the oranges treated for 25 d at 2.34 °C, as indicated in Table 7, demonstrating a 100% mortality for over 96,168 individuals of the most cold-tolerant stage of *Z. tau* in oranges. Both Navel and Valencia oranges treated at 2.34 °C for 25 d against *Z. tau* exceeded the required minimum assurance of 99% mortality at the 95% CL. Cold treatment of *Z. tau* on oranges combining Navel and Valencia cultivars at 2.34 °C for 25 d exceeded the required minimum assurance of 99.9968% mortality at the 95% confidence level and also passed the probit-9 level.

## 4. Discussion

In this study, the results indicated that third instar larvae of *Z. tau* were the most cold-tolerant developmental stage. This was consistent with other cold treatment research on *Z. tau* and *Z. cucurbitae* [17,18]. It was also observed that no survivors were detected among 106,204 estimated third instar larvae in Navel and Valencia oranges after being subjected to cold treatment of 23 d at 1.75 °C in the large-scale trials. This result aligned with the finding that the cold treatment of 22 d at ≤1.70 ℃ prevented *Z. tau* from developing to the adult stage with a high level of confidence [17]. However, if the cold treatment temperature increased to 2.34 °C, the exposure time should be extended to 25 d for 100% mortality of tested larvae, which was confirmed by larger-scale trials among 96,168 estimated third instar larvae in two cultivars of orange. These results met the requirement of no survivors found after post-treatment based on acute larval mortality as the quarantine treatment endpoint [22]. In this study, survival data for different developmental stages of *Z. tau* were analyzed using both probit and logit models. The results (see Table 3) indicate that the confidence intervals of the lethal time estimates derived from probit analysis were narrower than those obtained from the logit model, indicating a better fit of the probit model. However, the confidence intervals generated by the probit model remained relatively wide. For example, at 1.8 °C, the LT_99.9968_ for third instar larvae of *Z. tau* ranged from 17.7 d (minimum) to 23.1 d (maximum). This reflects the inherent difficulty of estimating extreme percentiles. In the present study, large-scale trials (sample size > 100,000 individuals) were conducted to empirically determine the duration required to achieve 100% mortality, which compensates for the uncertainty associated with model-based estimates. Therefore, for regulatory purposes, the proposed treatment durations (23 d and 25 d) are conservative and supported by substantial experimental data, rather than relying solely on model predictions.

Host preference and suitability tests have demonstrated that citrus is less susceptible to *Z. tau* than cucurbit fruit under both laboratory and field conditions [3,4]. The *Z. tau* populations tested in this study with detached oranges showed poor infestation levels, and this infestation would not be similarly utilized in attached fruits in commercial citrus groves [17]. The situation suggests that fruit damage may be an important factor for the infestation of *Z. tau* to oranges, similar to the infestation dynamics of *Z. cucurbitae* [23]. In order to avoid the factors of non-infestation of intact oranges or low infestation levels of puncture oranges, the hollowed-out fruit pulp method of artificial infestation of *Z. tau* on orange was used in this study. The method was confirmed as practical for obtaining a sufficient number of individuals during cold treatment trials. Artificial egg inoculation, as an alternative to natural oviposition, is commonly employed in cold treatment research on fruit flies in fruits [8,9,10,11,12,24]. The method described in this study—creating artificial cavities and directly inserting eggs into the fruit pulp—represents one such artificial inoculation technique. Larvae derived from artificial egg inoculation bypass the fruit’s natural physical and chemical barriers, such as the flavedo and albedo layers. Whether this practice affects larval cold tolerance, and to what extent such effects may influence results when large sample sizes are used, need further investigation.

Although the adults of *Z. tau* can be trapped by traps baited with Cue-lure in sweet orange orchards [25], at the time of writing, no documented observations of *Z. tau* infesting fruit in citrus production areas has been reported in the scientific literature, indicating that infestations in the field are likely rare [3,17,26]. Consequently, due to low host preference and performance in citrus, the occurrence of fruit infested in the pathway is diminished [17]. Furthermore, the systems approach, encompassing registered production sites, trapping requirements to re-maintain the fruit flies in lower prevent level, sorting and removing the defective fruits during packaging procedure, can effectively eliminate the risk of *Z. tau* from citrus fruits. Finally, the application of cold treatment, based on the result of 100% mortality observed at 1.8 °C for 22 d exposure for all 9108 estimated third instar larvae in this study, would provide an additional safety measure to mitigate the risk of *Z. tau* introduction and establishment during international citrus trades. In conclusion, as an additional safety measure, both the 22 d at ≤1.70 ℃ using a pupal endpoint reported by Dia et al. [17] and the 22 d at  1.8 ℃ using a larval endpoint according to this study support the application of the treatment of T107-o (18, 20, and 22 d at ≤0.56, 1.11, and 1.67 °C, respectively) which mitigates the risk of *Z. tau* introduction and establishment through the imported citrus pathway. However, for other susceptible hosts of *Z. tau*, the cold treatment schedule should extend to 23 d at 1.75 °C or 25 d at 2.34 °C with larval endpoint, according to the results in this study.

As insect populations may differ in thermal tolerance to ecologically relevant temperatures [5,27], *Z. tau* is a major pest species complex with taxonomic uncertainties. The cold tolerance of *Z. tau* third instar larvae from wild strains of Palampur (India), Fujian (China), and Baipayl (Bangladesh) and a laboratory strain from Fujian was compared, and the result showed that the populations from Fujian/wild and Palampur were the most cold-tolerant populations, although there was no significant difference in the 95% CL >99.9% mortality [17]. The wide populations of *Z. tau* used in our study were sourced from the Guangzhou area of China (latitude 24.1956° N, longitude 120.7105° E), adjacent to the Fujian area (latitude 26.0506° N, longitude 119.1423° E). This study provides confidence for plant protection organizations that a single cold treatment can achieve quarantine level control, regardless of future taxonomic divisions that may occur within the *Z. tau* complex. Future studies should evaluate the cold tolerance of *Z. tau* populations from different biogeographical regions, particularly those at higher risk of introduction via international trade.

The subgenus *Bactrocera* (*Zeugodacus*) has been elevated to genus status as taxonomic revision; this new genus includes important economic species such as *B.* (*Zeugodacus*) *cucurbitae* and *B*. (*Zeugodacus*) *tau* [28]. The cold tolerance has been well studied in some *Bactrocera* species including *B. dorsalis*, *B. tryoni* and *B. zonata* [9,10,11,12,29]. Some of these research results have been adopted by IPPC in ISPM 28, such as PT 16 (16 d at ≤3 ℃) for *B. tryoni* on *citrus sinensis* and PT 41 (18 d at ≤1.7 ℃) for *B. zonata* on *citrus sinensis* [13,15]. So far, published studies on cold tolerance in *Z. tau* and *Z. cucurbitae* remain limited [30]. Key findings from available research are summarized as follows: Myers et al. demonstrated that *Z*. (*Bactrocera*) *cucurbitae* had the highest cold tolerance among six *Bactrocera* species in their research (the other five species belong to subgenus *Bactrocera* (*Bactrocera*)), when tested as eggs and third instar in Navel orange or diet at 2 °C [31]. Follett et al. concluded that the inherent cold tolerance of *Z. cucurbitae* is equivalent to or higher than that of *C. capitata* [24]. Prakriti et al. reported that *Z. cucurbitae* exhibited higher cold tolerance levels than *Z. tau* [18]. The cold treatment schedule of the 22 d at ≤ 1.70 °C for *Z. tau* is considered equivalent to the highest severe cold treatment currently used for *Anastrepha ludens* on citrus [17]. However, the cold treatment schedule of 23 d at ≤ 2 °C for *C. capitata* on *Citrus reticulata* was adopted as PT 28 by ISPM 28 [32]. The cold treatment results of 23 d at 1.75 °C or the 25 d at 2.34 °C in this study showed that *Z. tau* has the highest cold tolerance among the other tephritid species for which cold treatment has been reported. Due to the two species of *Z. tau* and *Z. cucurbitae* existing in the same area and sharing the same hosts in some cases, further research on which one has higher cold tolerance are needed to assist authorities in importing countries in developing quarantine treatment schedules for the coexisting hosts of *Z. tau* and *Z. cucurbitae*.

## 5. Conclusions

The data reported in this paper showed that the third instar larval stage of *Zeugodacus tau* had the highest cold tolerance among all developmental stages. The method of artificial infestation with the hollowed-out fruit pulp was confirmed as a practical way to obtain a sufficient number of individuals for a cold tolerance study regardless of the susceptibility of the host. For citrus, a less susceptible host of *Z. tau*, the results supported that the application of the treatment of 22 d at ≤1.67 °C as an additional safety measure mitigated the risk of introduction and establishment of *Z. tau* through the imported citrus pathway. However, for other susceptible hosts of *Z. tau*, the cold treatment schedules need 23 d at 1.75 °C or 25 d at 2.34 °C with larval endpoint, according to the results of this study. These results showed that *Z. tau* exhibits the highest cold tolerance among the other tephritid species for which cold treatments have been reported.

## Figures and Tables

**Table 1:** Development rate of *Zeugodacus tau* in Navel orange at 25 °C and 22 °C.

Temp.	Stage	Percentage of Different Developmental Stage at Each Day After Egg-Oviposition							
		1 ^a^	2	3	4	5	6	7	8
25 °C	Egg	6.78 ± 6.83 ^b^							
	First instar	93.22 ± 6.83	40.09 ± 18.49	3.58 ± 2.39					
	Second instar		59.91 ± 18.49	83.97 ± 8.03	16.86 ± 1.36	2.29 ± 1.09			
	Third instar			8.56 ± 3.25	83.04 ± 1.23	96.81 ± 1.04	100.0 ± 0.0		
22 °C	Egg	100.0 ± 0.0							
	First instar		95.9 ± 2.7	22.2 ± 11.08	0.59 ± 1.03				
	Second instar		4.1 ± 2.7	77.9 ± 10.82	95.44 ± 3.16	30.1 ± 13.78	5.57 ± 3.71	0.63 ± 1.09	
	Third instar				3.97 ± 4.0	69.8 ± 13.78	94.43 ± 3.71	99.37 ± 1.09	100.0 ± 0.0

^a^ “1” means next day (about 24 h) after egg oviposition. ^b^ Data are expressed as mean ± SD of three replications.

**Table 2:** Mortality rates (mean ± SD) of different stage of *Zeugodacus tau* in Navel oranges subjected to cold treatment at 1.8 °C and 2.5 °C.

Temp.	Stage	No. of Treated	Exposure Time (d)				
			3	6	9	12	15
1.8 °C	Egg	3708	42.67 ± 7.02 a	70.71 ± 6.63 ab	98.64 ± 0.36 a	100 ± 0 a	100 ± 0 a
	First instar	2911	45.86 ± 15.47 a	76.57 ± 10.92 b	98 ± 1.23 a	99.29 ± 0.88 a	100 ± 0 a
	Second instar	3301	10.97 ± 4.26 b	55.19 ± 3.2 a	85.15 ± 6.23 b	96.42 ± 1.78 b	99.36 ± 0.31 a
	Third instar	3147	11.57 ± 1.22 b	62.61 ± 1.91 ab	80.24 ± 6.3 b	95.02 ± 1.49 b	98.35 ± 0.8 b
2.5 °C	Egg	4325	63.22 ± 8.2 a	73.19 ± 7.29 a	99.09 ± 0.25 a	100 ± 0 a	100 ± 0 a
	First instar	3709	55.47 ± 11.69 a	78.39 ± 8.85 a	98.63 ± 0.49 a	99.64 ± 0.5 a	100 ± 0 a
	Second instar	3574	29.72 ± 8.68 b	68.28 ± 9.52 a	93.21 ± 1.15 a	98.2 ± 0.97 a	99.81 ± 0.17 a
	Third instar	3286	12.38 ± 3.76 c	52.08 ± 1.85 b	74.69 ± 8.74 b	92.37 ± 4.02 b	96.93 ± 1.54 b

Note: within each column, values followed by different letters were significantly different based on lethal time of different stage trials (*p* < 0.05).

**Table 3:** Probit and logit analysis of different stages of *Zeugodacus tau* response to cold treatment at 1.8 °C and 2.5 °C.

Temperature	Type of Model	Stage	Slope	LT_99_ (95% CL) (Days)	LT_99.9968_ (95% CL) (Days)
1.8 °C	Probit	Egg	0.35 ± 0.01	10.5 (9.5, 11.8) b	15.2 (13.6, 17.6) b
		First instar	0.32 ± 0.01	10.7 (9.4, 12.8) b	15.9 (13.6, 19.8) b
		Second instar	0.33 ± 0.01	13.1 (12.1, 14.3) a	18.1 (16.5, 20.2) a
		Third instar	0.29 ± 0.00	14.1 (12.8, 15.9) a	19.9 (17.7, 23.1) a
	Logit	Egg	0.61 ± 0.01	11.4 (10.1, 13.3) b	20.9 (18.0, 25.5) b
		First instar	0.58 ± 0.01	11.4 (9.9, 14.0) b	21.3 (17.7, 27.6) b
		Second instar	0.60 ± 0.01	13.7 (12.7, 14.9) a	23.2 (21.1, 26.1) ab
		Third instar	0.52 ± 0.01	14.8 (13.4, 16.8) a	25.9 (22.8, 30.5) a
2.5 °C	Probit	Egg	0.27 ± 0.01	11.1 (9.5, 14.1) bc	17.4 (14.4, 23.3) b
		First instar	0.30 ± 0.01	10.6 (9.3, 12.6) c	16.2 (13.9, 20.0) b
		Second instar	0.31 ± 0.01	12.0 (11.1, 13.3) b	17.4 (15.8, 19.6) b
		Third instar	0.26 ± 0.00	15.5 (14.3, 17.2) a	22.0 (19.9, 24.8) a
	Logit	Egg	0.47 ± 0.00	12.2 (10.1, 16.4) b	24.5 (19.3, 35.3) b
		First instar	0.54 ± 0.01	11.4 (9.8, 14.0) b	22.1 (18.2, 28.8) b
		Second instar	0.56 ± 0.01	12.7 (11.7, 14.0) b	23.0 (20.7, 26.1) b
		Third instar	0.46 ± 0.01	16.6 (15.2, 18.5) a	29.3 (26.1, 33.6) a

Note: within each column, values followed by different letters were significantly different based on lethal time of different stage trials (*p* < 0.05).

**Table 4:** Small-scale trials showing the mortality rates of third instar of *Zeugodacus tau* in Navel orange of cold treatment at 1.8 °C.

Treatment Duration	NR ^a^	Control		Treated			
		No. of Fruits	No. of Survivors	No. of Fruits	EN ^b^	No. of Survivors ^c^	Mortality (%)
17 days	3	30	3036	90	9108	15 ± 4	99.50 ± 0.15
19 days	3	30	3036	90	9108	7 ± 7	99.78 ± 0.19
21 days	3	30	3036	90	9108	1 ± 2	99.97 ± 0.06
22 days	3	30	3036	90	9108	0	100.0
23 days	3	30	3036	90	9108	0	100.0

**^a^** NR: numbers of replications, ^b^ EN estimated No. of treated third instar = No. of survivors in control × (No. of treated fruits/No. of control fruits), ^c^ Each treatment contains data are expressed as mean ± SD of three replications.

**Table 5:** Small-scale trials showing the mortality rates of third instar of *Zeugodacus tau* in Navel orange of cold treatment at 2.5 °C.

Treatment Duration	NR ^a^	Control		Treated			
		No. of Fruits	No. of Survivors	No. of Fruits	EN ^b^	No. of Survivors ^c^	Mortality (%)
19 days	3	30	2133	90	6399	18 ± 16	99.09 ± 0.36
21 days	3	30	2133	90	6399	8 ± 3	99.60 ± 0.22
23 days	3	30	2133	90	6399	1 ± 1	99.96 ± 0.07
24 days	3	30	2133	90	6399	0	100.0
25 days	3	30	2133	90	6399	0	100.0

**^a^** NR: numbers of replications, ^b^ EN estimated No. of treated third instar = No. of survivors in control × (No. of treated fruits/No. of control fruits), ^c^ Each treatment contains data are expressed as mean ± SD of three replications.

**Table 6:** Large-scale trials showing the mortality rates of third instar of *Zeugodacus tau* in two orange cultivars of cold treatment for 23 days at 1.75 °C.

Cultivars	Replications	Control		Treatment			
		No. of Fruits	No. of Survivors	No. of Fruits	EN ^a^	No. of Survivors	Mortality (%)
Navel	1	50	6857	200	27,428	0	100
	2	50	3415	200	13,660	0	100
	3	50	4052	200	16,208	0	100
	Total	150	14,324	600	57,296	0	100
Valencia	1	50	4731	200	18,924	0	100
	2	50	4219	200	16,876	0	100
	3	50	3277	200	13,108	0	100
	Total	150	12,227	600	48,908	0	100

^a^ EN estimated No. of treated third instar = No. of survivors in control × (No. of treated fruits/No. of control fruits).

**Table 7:** Large-scale trials showing the mortality rates of third instar of *Zeugodacus tau* in two orange cultivars of cold treatment for 25 days at 2.34 °C.

Cultivars	Replications	Control		Treatment			
		No. of Fruits	No. of Survivors	No. of Fruits	EN ^a^	No. of Survivors	Mortality (%)
Navel	1	50	4612	200	18,448	0	100
	2	50	4228	200	16,912	0	100
	3	50	3852	200	15,408	0	100
	Total	150	12,692	600	50,768	0	100
Valencia	1	50	3358	200	13,432	0	100
	2	50	4012	200	16,040	0	100
	3	50	3982	200	15,928	0	100
	Total	150	12,352	600	45,400	0	100

^a^ EN estimated No. of treated third instar = No. of survivors in control × (No. of treated fruits/No. of control fruits).

## Data Availability

The original contributions presented in this study are included in the article. Further inquiries can be directed to the corresponding authors.
